# Influence of Cover Crop Root Functional Traits on Sweet Potato Yield and Soil Microbial Communities

**DOI:** 10.3390/microorganisms13030471

**Published:** 2025-02-20

**Authors:** Xinyi Chen, Jie Zhang, Wangbiao Xia, Yangyang Shao, Zhirong Liu, Jian Guo, Wenjing Qin, Li Wan, Jia Liu, Ying Liu, Juntong Zhang

**Affiliations:** 1 Jiangxi Provincial Engineering Research Center for Seed-Breeding and Utilization of Camphor Trees, School of Soil and Water Conservation, Nanchang Institute of Technology, Nanchang 330200, China; y99_xinchen@163.com (X.C.); wbxia0616@163.com (W.X.); yang871775505@163.com (Y.S.); zr15170856270@163.com (Z.L.); ggjian2016@163.com (J.G.); 2 Institute of Soil and Fertilizer & Resource and Environment, Jiangxi Academy of Agricultural Sciences, Nanchang 330200, China; qinwenjing033@jxaas.cn (W.Q.); wanli@jxaas.cn (L.W.); liujia@jxaas.cn (J.L.); 3 Jiangxi Yichun Selenium Resources Development and Utilization Center, Yichun 336000, China; ycfxcy@126.com; 4 Hebei Institute of Product Quality Supervision and Inspection, Shijiazhuang 050227, China

**Keywords:** cover crops, resource-acquisitive, resource-conservative, microbial community, species richness, network complexity

## Abstract

The symbiotic relationship between cover crops and soil microorganisms is closely linked to nutrient cycling and crop growth within agroecosystems. However, how cover crops with different root functional traits influence soil microbial communities, soil properties, and crop yields has remained understudied. This study assessed the root traits of hairy vetch (HV) and rapeseed (RP), along with soil properties, sweet potato yield, and microbial enzyme activity under red soil dryland conditions. High-throughput sequencing was also employed to characterize the diversity, composition, and network structure of soil bacterial and fungal communities. According to the plant economic spectrum theory and our research results on plant root traits, HV can be identified as a resource-acquisitive cover crop, and RP treatment can be identified as a resource-conservative cover crop. Although RP treatment did not significantly increase the sweet potato yield, the increase rate reached 8.49%. Resource-conservative cover crops were associated with increased pH, SOC, and TP, which enhanced bacterial species diversity and boosted the populations of Chloroflexi and Alphaproteobacteria. In contrast, resource-acquisitive cover crops promoted the proliferation of Gammaproteobacteria. Network analysis indicated that resource-conservative cover crops facilitated network complexity through intensified intra-community competition. Resource-acquisitive cover crops enhanced the stability of microbial communities. Collectively, these findings underscore the distinct advantages of cover crops with varying root functional traits in shaping soil microbial communities. Appropriate cover crop rotations can effectively regulate microbial communities and hold the potential to enhance crop yield.

## 1. Introduction

Agricultural soils confront the dual challenge of sustaining high crop yields and safeguarding vital ecosystem services, underscoring an urgent need for sustainable agricultural practices [1]. Employing diverse cover crops can boost crop yields and soil health alike, offering a means to enrich agroecosystem diversity [2]. Although numerous studies have documented the yield-increasing effects of cover crops and staple crop rotations, scant research has addressed soil function in terms of biodiversity.

Soil microbial communities are integral to soil ecosystems, playing a crucial role in ecological functions, including plant productivity enhancement [3,4,5], nutrient cycling facilitation [6], organic matter decomposition, and plant pathogen regulation [7]. Incorporating cover crops into traditional crop rotation systems has been demonstrated to enhance agricultural sustainability, offering a multitude of ecological benefits [8]. Cover crops influence soil microbial community composition and abundance through direct plant–microbe interactions and indirectly by altering soil structure and its physical and chemical properties [9,10]. For example, reintroducing legumes to fields can stimulate eutrophic microorganism development, increase soil microbial activity, and significantly increase the number and diversity of nitrogen-fixing microbes [11]. In contrast, cruciferous plants, characterized by a high C/N ratio and complex, hard-to-decompose organic matter [2], promote the growth of oligotrophic microorganisms dependent on complex organic matter for energy [12]. Planting cereal crops has been found to increase soil microbial biomass carbon (MBC) and microbial biomass nitrogen (MBN), subsequently inducing shifts in soil fungal and bacterial communities [13]. The findings indicate that the selection of cover crop species is a pivotal determinant in the formation of soil microbial communities.

The plant economic spectrum theory provides a valuable framework for trait-based ecological studies. Plants exhibit diverse functional traits concerning resource acquisition, growth rate, lifespan, and reproductive strategy, and show significant covariations among traits for resource acquisition and conservation [14,15]. Plant species at the resource-acquisitive end of the spectrum have well-developed root systems, increased root hair density, and high nitrogen content, which augment synergies between tufted mycorrhizal fungi and nitrogen-fixing bacteria, enhancing the soil nitrogen fixation potential [16]. Conversely, resource-conservative species, like oilseed rape, with higher cellulose and lignin levels [2] and greater rooting depth, rely on mycorrhizal fungi symbiosis for efficient resource acquisition [17]. The current research on plant strategies has focused on soil property and fertility changes due to cover crop tillage. Yet, the literature lacks comprehensive insights into how cover crop root traits affect soil microbial communities.

Red soil drylands in southern China are a vital agricultural resource; however, they face degradation processes such as erosion [18], acidification [19], and a decline in soil fertility and ecological functions [19], which limit the productivity of these soils. The introduction of diverse cover crops provides a pathway to sustainable agricultural development in red soil drylands, enhancing soil microbial community composition and maintaining its multifunctional capabilities [3]. However, the effects of cover crops on the microbiome of dryland red soils differ significantly among species [9], such as sorghum and cereal rye, influence plant and soil functional outcomes through the manipulation of root exudates. Their findings illustrated that microbial metabolic patterns were unique in response to cover crop exudate inputs over time [20]. Therefore, the integration of cover crops into red soil ecosystems requires a careful consideration of the functional traits of these crops. This is essential because these traits significantly influence the interaction between the crops and the soil microbiome, thereby affecting soil health and productivity. This study investigated the influence of functional traits from two cover crops of different families on microbial community characteristics in dryland soils. This study utilized a winter cover crop rotation followed by a summer sweet potato cultivation to establish a scientific basis for enhancing soil functionality and advancing sustainable agricultural practices in red soil regions.

## 2. Materials and Methods

### 2.1. Study Site Description and Initial Soil Conditions

This study was conducted at the Dafangshan experimental site, located in Jinxian County, Jiangxi Province (116°35′11″ E, 28°10′59″ N). The region features a subtropical monsoon humid climate, with a mean annual temperature of 17.7 °C and an annual precipitation of about 1727 mm. The pH of red soil in Jinxian County, which was 5.9 in 1982, declined to 4.8 by 2017, reflecting significant soil acidification. The experimental site’s soil is derived from Quaternary red clay, classifying it as red soil. Before planting cover crops, the topsoil (0–20 cm) pH was 4.68. Moreover, the baseline nutrient levels of the soil organic matter (SOC), total nitrogen (TN), total phosphorus (TP), alkaline dissolved nitrogen (AN), and available phosphorus (AP) were 12.31 g/kg, 1.11 g/kg, 0.55 g/kg, 0.55 g/kg, and 31.8 mg/kg, respectively.

### 2.2. Experimental Design

This study was initiated in 2022, after the late October sweet potato harvest. Our study featured three treatments: (1) a winter fallow control (CK), (2) hairy vetch (*Vicia villosa* Roth) (HV), and (3) rapeseed (*Brassica napus* L.) (RP). The treatments were arranged in a randomized block design with 6 replications. The experimental area, measuring 30 m^2^, was configured as 6 m × 5 m. Cover crops were sown using a broadcast method on 25 October 2022. The sowing rates were 40 kg/hm^2^ for hairy vetch and 10 kg/hm^2^ for rapeseed. These sowing rates considered the crops’ growth characteristics, competitive interactions, resource requirements, and experimental objectives, aligning with previous studies and local farming customs. The control plots were maintained as fallow land during the winter season, without herbicides or artificial weed control measures. The winter cover crops matured without irrigation or fertilizer supplementation during the growing season.

In early April 2023, during the flowering phase of cover crops, we mechanically incorporated all cover crops into the soil to a depth of 10–20 cm, replicating the winter treatment method. Subsequently, in early May, sweet potatoes were transplanted with a ridge spacing of 1 m, plant spacing of 20 cm, and a planting density of 50,000 plants per hectare. Following local agricultural norms and production practices, sweet potatoes were fertilized with the following annual application rates: N at 180 kg/hm^2^, P_2_O_5_ at 90 kg/hm^2^, and K_2_O at 270 kg/hm^2^. The base fertilizer consisted of one-third N fertilizer, with the remaining two-thirds applied as a top-dressing during the small bell mouth stage of corn. Urea, containing 46% N, was used as the nitrogen fertilizer; calcium–magnesium–phosphorus fertilizer, containing 12% P_2_O_5_, was used as the phosphorus fertilizer; and potassium chloride, containing 60% K_2_O, was used as the potash fertilizer, with phosphorus and potash applied as basal fertilizers. All fertilizers were purchased from a local agricultural company (Nanchang City, China).

### 2.3. Soil Sampling Methodology and Functional Traits of Cover Crops

Soil samples (500 g/sample) were collected during the sweet potato seedling stage in late May 2023. The soil samples were extracted from a 0–20 cm depth in each plot using a soil core sampler (diameter of 2.0 cm) following the 5-point method [21], and then mixed thoroughly to form one replicate sample, yielding a total of 18 samples (3 treatments × 6 replicates). Post-collection, the soil samples were promptly returned to the laboratory, where they were cleaned of impurities, sifted through a 2 mm sieve, and then each sample was split into two portions: one air-dried for soil physical and chemical property assessment, and the other stored at −20 °C for microbial community analysis.

The functional traits of each cover crop were measured at the peak flowering stage (mid-March 2023). Specifically, five representative cover crop plants with relatively uniform growth were excavated from each plot and transferred to the laboratory for thorough cleaning. The cleaned root samples were scanned using a root scanner (EPSON LA2400 Scanner, Seiko Epson, Suwa, Japan) and analyzed with WINRHIZO software (Regent Instruments Inc., Quebec City, QC, Canada; Version 2017b) to determine the following root characteristics: length (cm), volume (cm^3^), tip number (tips), surface area (cm^2^), and diameter (mm). Following scanning, all root samples were dried at 70 °C for 48 h, after which their carbon (C) and nitrogen (N) contents were quantified following manual grinding.

### 2.4. Soil Analyses

We selected five pivotal soil ecological function indicators to evaluate the soil ecosystem’s service capacity: SOC (soil organic carbon), TN (total nitrogen), TP (total phosphorus), AP (available phosphorus), and AN (available nitrogen). These indicators play dual roles in supporting and regulating the ecological system, and they encompass a range of functions, such as nutrient cycling, soil properties, and fertility [22]. The soil pH was measured at a 1:2.5 soil-to-water ratio using a pH meter (FE28-Standard, METTLER-TOLEDO, Zurich, Switzerland) [23]. The SOC concentrations were determined via dichromate oxidation, subsequent to titration with ferrous sulfate. The TN was quantified using the Kjeldahl method, which entails digestion and distillation processes [24]. The TP was measured utilizing the alkali fusion–Mo-Sb colorimetric method. The AP was determined by combining sodium bicarbonate (NaHCO_3_) leaching with the Mo-Sb colorimetric method, and the AN was quantified using the alkali hydrolysis technique [25].

Soil enzyme activities associated with the carbon (C), nitrogen (N), and phosphorus (P) cycles were quantified using fluorometric methods following the protocol outlined by Marinari [26]. Enzymes involved in the C cycle included α-1,4-glucosidase (AG), β-1,4-glucosidase (BG), β-xylosidase (XYL), and β-D-cellobiohydrolase (CB). Those linked to the N cycle comprised β-1,4-N-acetylglucosaminidase (NAG) and leucine aminopeptidase (LAP), while acid phosphatase (ACP) was measured as an indicator of the P cycling activity.

### 2.5. DNA Extraction and Amplicon Sequencing

A quantity of 0.5 g of the soil samples was weighed, and the DNA was extracted using the FastDNA^®^ SPIN Kit for Soil (MP Biomedicals, Santa Ana, CA, USA), in accordance with the manufacturer’s protocol. The concentrations of the extracted DNA were then determined using a NanoDropTM 2000 Spectrophotometer (Thermo Scientific, Waltham, MA, USA) by calculating the 260/280 nm absorbance ratio. The amplification of bacterial 16S rRNA gene fragments was achieved using primers 341F (5′-CCTACGGGGNGGCWGCAG-3′) and 806R (5′-GGACTACHVGGGGTATCTAAT-3′). Furthermore, the primer pair ITS1F (5′-CTTGGTCATTTAGAGGAAGTAA-3′) and ITS2R (5′-GCTGCGTTCTTCATCGATGC-3′) was used to target the fungal internal transcribed spacer (ITS) region for community analysis [27,28]. The PCR products underwent bidirectional high-throughput sequencing on the Illumina MiSeq PE250 platform (Shanghai Lingen Biotechnology Co., Ltd., Shanghai, China).

Utilizing the QIIME2 pipeline, a comprehensive analysis of the amplified sequences was conducted. Initially, the ’cutadapt’ tool was employed to eliminate the primer sequences, ensuring the accuracy of subsequent analysis. Subsequently, the ’Demux’ plugin was utilized for quality control on the original sequences, with sequences averaging below 25 on quality scores and those shorter than 50 bp being removed. The DADA2 algorithm was then applied to merge the quality-controlled sequences, classify the amplicon sequence variants (ASVs) at 99% similarity, and remove redundancies. Finally, bacterial sequences were compared and classified against the Silva 138.1 database, thereby deepening our insight into the samples’ microbial diversity. The SILVA database (available at the website arb-silva.de; accessed on 7 June 2023.) was used to assign taxonomic designations to bacterial ASV.

### 2.6. Data Analysis

All statistical analyses were conducted in R Studio (version 4.4.2; R Core Team 2024), and the “ggplot2” package was used to make figures to visualize the results. All significant differences were determined by one-way analysis of variance (ANOVA) followed by Fisher’s least significant difference (LSD test) method at a significance level of α = 0.05. First, to investigate the differences in the functional traits among the cover crops, principal component analysis (PCA) was carried out by using “FactoMineR”. Second, the “vegan” R package was utilized to assess β-diversity and α-diversity indices across various treatments. The number of observed amplicon sequence variants (ASVs) in each sample was selected as a measure of α-diversity to examine changes in the α-diversity of soil bacteria and fungi associated with different root traits. Third, canonical analysis of principal coordinates (CAP) was executed using the capscale() function in the “vegan” package to explore the effects of cover crops’ functional traits on the structure and function of bacterial communities.

Manhattan plots were generated using the “edgeR” and “dplyr” packages to compare soil bacteria from the CK treatment with those from the HV and RP treatments, respectively [29]. Differential ASV abundance and taxa were analyzed using Wilcoxon rank sum tests, and the corresponding *p*-values were corrected for multiple tests using a FDR set to 0.05. ASVs above the significance threshold were considered significantly enriched. Finally, comprehensive analysis of co-occurrence networks was conducted to elucidate variations in the microbial network structure and core taxa. The dataset was meticulously filtered prior to network construction to eliminate ASVs with infrequent appearances, with the retention of those present in at least half of the samples. Using the subgraph() function in the “igraph” package, three subnetworks (representing the networks of individual samples) were extracted from the bacteria and fungal global network, respectively, based on representative nodes of each sample. Correlation coefficients were then computed using the corAndPvalue() function from the “WGCNA” package. The *p*-values underwent a Bonferroni correction, and irrelevant correlations were filtered with the criteria of |R| > 0.6 and *p* < 0.05, leading to network generation. The diversity(), gsize(), and transitivity() functions from the “igraph” package were employed to determine the number of nodes, connections, and average clustering coefficients, facilitating the characterization of the network’s topological features. Network visualizations were created via the plot() function for network representation, and all additional plots were generated utilizing the “ggplot2” package.

## 3. Results

### 3.1. Cover Crop Functional Traits and Ecological Strategies

**Figure 1:**
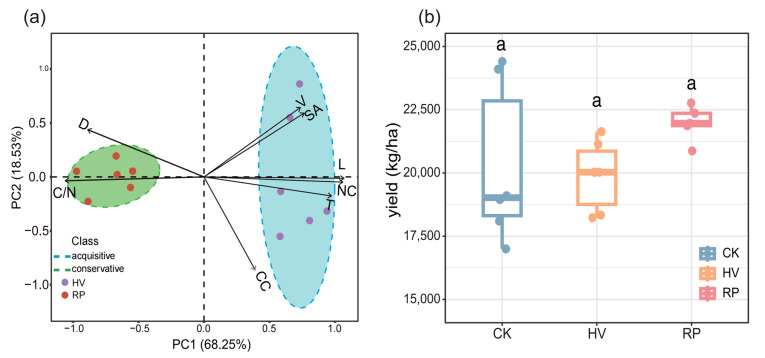
(**a**) Principal component analysis (PCA) of functional traits for two distinct cover crops. The figure displays two distinct clusters representing cover crop ecological strategies. One species on the left represents the acquisitive end of the plant economic spectrum, while the other on the right represents the conservative end. The green and blue ovals represent the conservative and acquisitive strategies, respectively. HV, hairy vetch; RP, rapeseed; D, root diameter; C/N, carbon-to-nitrogen ratio; CC, carbon content; NC, nitrogen content; V, volume; SA, surface area; T, number of root tips; L, length. (**b**) Variations in sweet potato yield under different cropping treatments. The same lowercase letter above the box indicates no significant difference (*p* > 0.05).

Principal component analysis (PCA) disclosed the economic spectrum traits of the two cover crops, accounting for 68.25% and 18.53% of the total variation with the first and second principal components, respectively (Figure 1). The RP treatment displayed root traits indicative of conservative strategies, featuring a large root diameter and a high C/N ratio (Table 1). Conversely, the HV treatment exhibited root traits consistent with the acquisition end of the economic spectrum, marked by high nitrogen content, a large surface area, an extensive root system, and a high number of root tips (Table 1). Based on these findings, this study categorized HV as an acquisition-type cover crop and RP as a conservation-type cover crop.

### 3.2. Sweet Potato Yield and Soil Properties

Sweet potato yield ranged from 13,650 kg/ha to 24,400 kg/ha. Compared with the winter fallow treatment, although the increase in sweet potato production did not reach a significant level, there were different degrees of yield improvement under different cover crop amendments (*p* > 0.05). The highest sweet potato yields were 21,635 kg/ha under the HV treatment and 22,360 kg/ha under the RP treatment, reaching an increase rate of 8.49% (Figure 1b).

**Figure 2:**
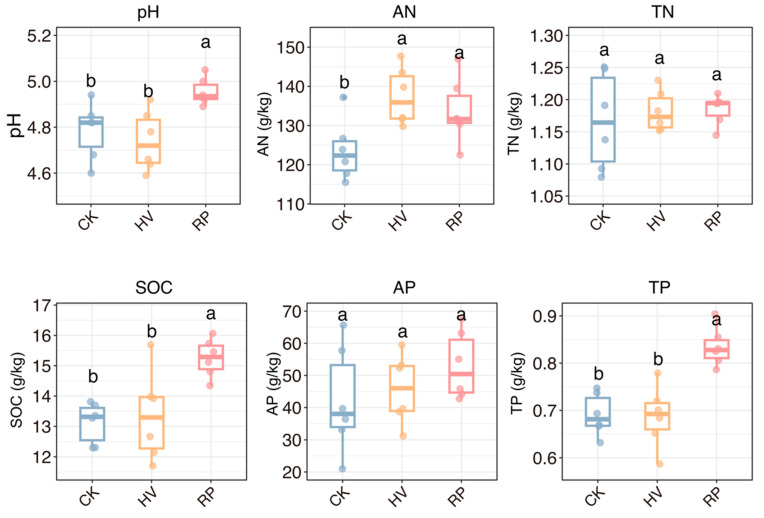
CK, winter fallow; HV, hairy vetch; RP, rapeseed. AN, available nitrogen; TN, total nitrogen; SOC, soil organic carbon; AP, available phosphorus; TP, total phosphorus. Different letters within the same row indicate significant differences among the treatments (*p* < 0.05).

The chemical properties of dryland red soil were notably altered following various cover crop intercropping treatments (Figure 2). The soil pH did not differ significantly among the treatments; however, the RP treatment elevated the pH to 4.95, whereas the HV treatment decreased it to 4.74 (Table A1). This suggests that the cultivation of RP contributed to soil acidification. The HV treatment primarily influenced the soil AN levels. Compared to the CK, the RP treatment markedly enhanced the soil SOC and TP by 19.13% and 20.29%, respectively (*p* < 0.05) (Table A1). The HV treatment, however, did not achieve statistical significance.

### 3.3. Cover Crop Amendments Led to Alterations in Soil Microbial Diversity, Community Composition, and Keystone Species

**Figure 3:**
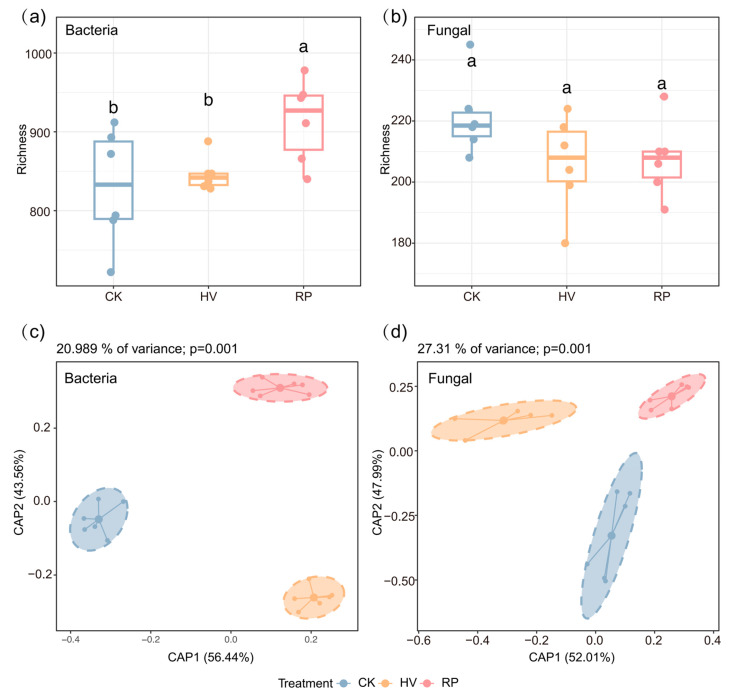
Influence of various cover crops on microbial community diversity and composition. The impact of different cover crop treatments on bacterial (**a**) and fungal (**b**) richness indices is depicted. CAP biplots display the differences in bacterial (**c**) and fungal (**d**) consortia associated with various cover crops. Cover crops accounted for 20.989% of the total variance in bacterial communities and 27.31% in fungal communities. Different lowercase letters above the boxes indicate significant difference among the treatments (*p* < 0.05).

Compared with the winter fallow treatment, rapeseed–sweet potato rotation increased the soil CB, LAP, and ACP activity significantly (Table A1). Statistical analysis revealed that diverse cover crop treatments significantly influenced bacterial diversity within the dryland red soil (*p* < 0.05; Figure 3a). Notably, the incorporation of RP led to a significant enhancement in soil bacterial species richness compared with the CK. In terms of fungal abundance, the results indicate no statistically significant differences among the treatments. Nevertheless, both the HV and RP treatments displayed a trend toward reduced fungal abundance relative to the CK (Figure 3b). This implies that although cover crop treatments did not significantly alter the fungal populations, the HV and RP treatments exhibited notable reductions in fungal abundance relative to the CK, necessitating further exploration into the potential reasons behind these trends.

Canonical analysis of principal coordinates (CAP) disclosed that microbial consortium under the cover crop treatments markedly diverged from those of the CK treatment, explaining 20.989% and 27.3% of the variation in the bacterial and fungal communities, respectively, *p* = 0.001; Figure 3c). Despite no significant differences in the fungal α-diversity, variance analysis of the community structure demonstrated that the soil fungal communities varied significantly among the cover crop treatments (Figure 3d). Notably, significant distinctions in both the soil bacterial communities were evident in CAP1 and CAP2.

**Figure 4:**
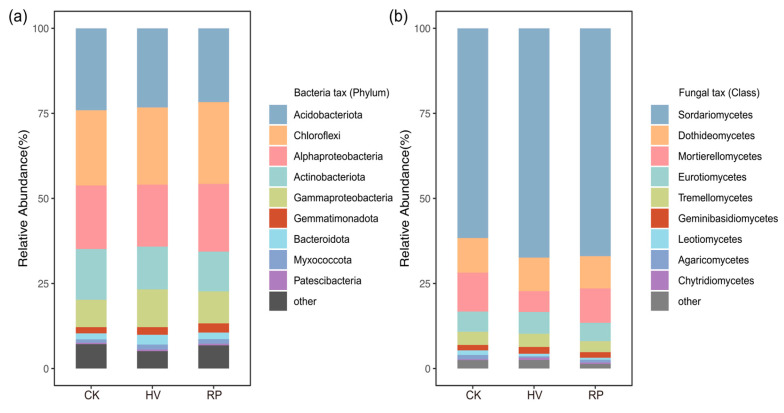
Relative abundance of soil bacterial communities at the phylum (**a**) and fungal (**b**) communities at the class level under different treatments.

High-ranking bacteria were selected at the phylum level and fungi were selected at the class level. Acidobacteriota, Chloroflexi, Alphaproteobacteria, Actinobacteriota, and Gammaproteobacteria were the most abundant phyla of bacteria (Figure 4a). At the class level of fungi, Sordariomycetes, Dothideomycetes, Mortierellomycetes, Eurotiomycetes, and Tremellomycetes were considered the dominant bacteria in this study (Figure 4b).

**Figure 5:**
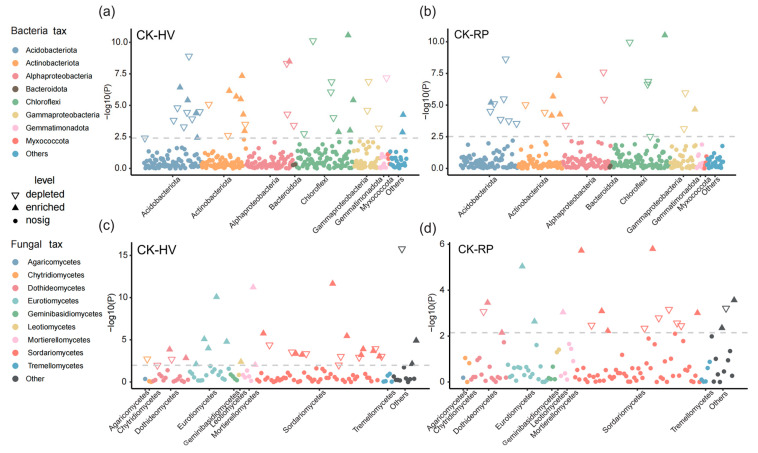
Manhattan plots of ASV enrichment in the HV or RP treatments relative to the CK treatment. The plots highlight bacterial species differences between the CK and HV (**a**) and the CK and RP (**b**) treatments. The enrichment of ASVs are shown in the HV (**c**) and RP (**d**) treatments in contrast to the CK treatment at the fungal level. Each dot or triangle represents an individual ASV, with the colors indicating different phyla. The solid triangles denote the ASVs enriched in the CK treatment, whereas the empty triangles represent those enriched in the HV or RP treatments. The circles positioned below the dashed line signify noise (FDR adjusted *p* < 0.05, Wilcoxon rank sum test). CPM, counts per million.

Subsequently, we analyzed soil microbiota variations under the HV and RP treatments at the ASV level. Differential abundance analyses revealed that cover crop amendments enriched ASVs across various bacterial phyla (Figure 5a,b). Notably, the legume cover crop amendment (HV) affected the abundance of 40 ASVs, and cruciferous cover crop amendment (RP) affected 25 ASVs. Specifically, three ASVs from the Gammaproteobacteria class were consistently enriched by the HV treatment (Figure 5a). In contrast, Chloroflexi and Alphaproteobacteria were the phyla and classes with the highest number of RP-enriched ASVs (Figure 5b). For soil fungi, Sordariomycetes abundance increased under both the HV and RP treatments (Figure 5c,d). Mortierellomycetes richness decreased under the HV treatment compared to the CK. The HV treatment resulted in an enrichment of Chytridiomycetes (Figure 5c).

### 3.4. Co-Occurrence Network Structures of Soil Bacteria and Fungi

**Figure 6:**
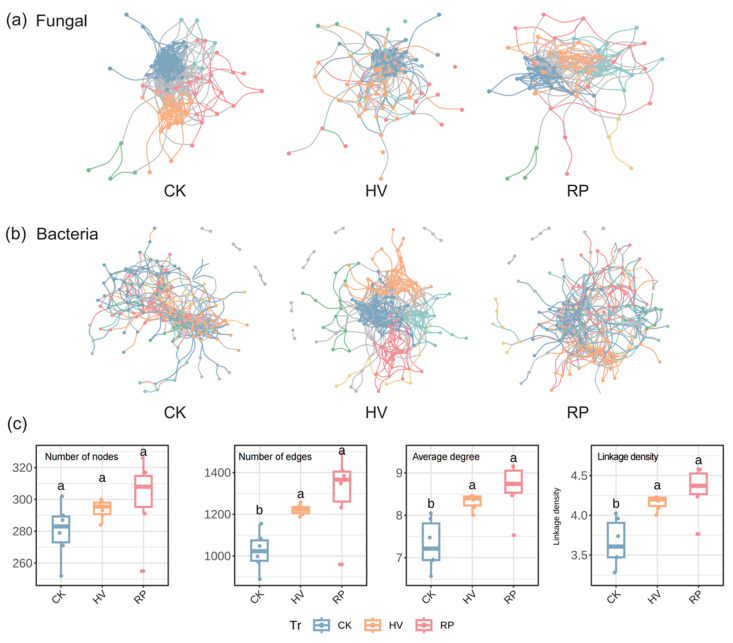
Symbiotic network structures in different cover crops with diverse functional traits. (**a**) Co-occurrence network patterns of soil fungi. (**b**) Co-occurrence network patterns of soil bacteria. Distinct colors represent different ecological clusters, and circle sizes correspond to the relative abundance of ASVs. (**c**) Topological characteristics of soil bacteria co-occurrence networks, including the number of nodes, number of edges, average degree, and linkage density. Different lowercase letters above the boxes indicate significant difference among the treatments.

To explore potential microbial interactions under different root trait cover crop treatments, we constructed three bacterial and three fungal meta-networks, respectively (Figure 6a,b). Within these networks, each node represents an ASV, and the edges represent species interactions, collectively reflecting the network’s complexity.

For the soil bacteria, the node counts for the three treatments were as follows: 280 for CK, 293 for HV, and 300 for RP (Table 2, Figure 6b). The total numbers of edges were 1024 for CK, 1221 for HV, and 1304 for RP. The average degree values were 3.66 for CK, 4.17 for HV, and 4.35 for RP (Table 2). Connection density, a measure of network complexity, was 7.31 for CK, 8.33 for HV, and 8.69 for RP (Table 2). Compared with the winter fallow treatment, all cover crop treatments markedly enhanced the number of network edges, average degree, and linkage density (Figure 6c). The number of nodes in various cover crop treatments exceeded that of the CK. Positive correlations among the soil microorganisms were predominant across the three treatments, at 71.94% for CK, 66.85% for HV, and 60.48% for RP (Table 2). Notably, the negative correlation among the soil microorganisms was more pronounced in the RP treatment compared to the other treatments.

In our investigation of soil fungi, the HV and RP treatments led to a decrease in both the node and edge counts within the network (Table 2, Figure 6a). For RP, the network had 72 nodes, 352 edges, an average degree of 4.89, and a link density of 9.78 (Table 2). Relative to the CK, the HV treatment’s fungal network exhibited a reduction in the number of nodes and edges. However, both the average degree and link density increased, suggesting more connections among fungi. Meanwhile, the RP treatment’s network was less complex but exhibited higher competition. Positive correlations prevailed among the soil fungi across the three treatments, with 67.2% for CK, 59.3% for HV, and 54.0% for RP (Table 2). In summary, both types of cover crops predominantly exhibited positive correlations among the soil microorganisms, and their cultivation positively influenced the structure of soil microbial networks.

## 4. Discussion

Our study demonstrates that different cover crop amendments exerted a significant influence on soil properties and sweet potato yield. While both treatments resulted in improved sweet potato yield, the resource-conservative cover crop treatment (RP) achieved a higher yield (Figure 1b). Compared to the CK, the RP treatment notably enhanced several key soil properties, including the pH level, AN, SOC, and AP content (Figure 2). In contrast, the HV treatment resulted in a decrease in pH (Figure 2), suggesting that the cultivation of hairy vetch effectively mitigated soil acidification. HV, as a leguminous species, substantially increased the AN content following its incorporation into the soil (Figure 2) [30]. Resource-conservative cover crop root systems, characterized by high tissue carbon content and thick root diameters (Table 1), can break red soil crust [31,32] and enhance soil aeration and water retention [33]. The resource-conservative cover crop (RP) residues were rich in recalcitrant carbon sources, like lignin and cellulose, and root exudates with elevated organic acid concentrations [34]. This is corroborated by our results (Table A1, Figure 2), showing significantly increased soil CB, LAP, and ACP activity under the RP treatment. The improvements in the soil quality indicate that rapeseed–sweet potato rotation system had better performance in increasing yield in red soil drylands.

Microbial diversity is an important indicator of soil health and nutrient cycling. Our study revealed a predominant link between soil microbial α-diversity and the type of cover crop applied (Figure 3a). The addition of RP significantly increased the species richness of soil bacteria compared to the CK (Figure 3a). However, no significant differences were observed in the fungal abundance between the treatments (Figure 3b). The integration of cover crops into the soil led to increased nutrient levels and improved microenvironments, potentially explaining the observed increase in the soil bacterial α-diversity [35,36]. For example, significantly higher soil SOC and TP concentrations appeared under cover crop fill-in practices than in the CK (Figure 2) [12,37]. In addition, the resource-conservative cover crop provided an optimal soil structure, promoting microbial colonization and dispersal, and thereby fostering a diverse range of microorganisms (Figure 1) [6]. However, the fungal α-diversity was notably reduced (Figure 3b), contrasting with the findings of Schmidt [38]. Some studies have shown that soil fungal diversity may be negatively correlated with the AN [39,40]; they compete for nutrients, resulting in low fungal diversity. From these long-term cover crop rotation studies, we can predict that cover crop selection strategies with different root traits can maintain soil diversity and provide long-term benefits for the sustainable development of red soil drylands.

This study clarifies the influence of cover crop root traits on soil microbial community structure and highlights the importance of microbial community growth strategies. The selection pressures, stemming from tillage methods and specific crop cultivation, result in significant shifts in soil microbial community compositions [41]. This study observed that both the bacterial and fungal community compositions were influenced by crop selection (Figure 3c,d), aligning with previous research [3]. This discrepancy may be because various cover crop types can selectively enrich specific microbial species via their root secretions and modify plant–microbial interactions [20,42], ultimately altering the microbial community structure and function [20]. Therefore, activities triggered by host-specific root exudates are pivotal in shaping distinct root-associated microbiomes among different plant species [43]. Soil bacterial ASVs affiliated with Gammaproteobacteria were notably enriched under the resource-acquisitive cover crop (HV) treatment (Figure 4a and Figure 5a). This enrichment is likely due to the readily decomposable carbon source released by HV, leading to the proliferation of eutrophic groups [44] that rapidly utilize carbon sources for growth rather than storage [45]. This pattern could be explained by the absence of a substantial increase in the soil SOC under the resource-acquisitive cover crop treatment. Conversely, the phyla and classes showing the most significant enrichment under the resource-conservative cover crop treatment were Chloroflexi and Alphaproteobacteria (Figure 4a and Figure 5b). This enrichment is likely due to resource-conservative cover crop (RP) residues, which are rich in recalcitrant carbon sources, like lignin and cellulose, and root exudates with elevated organic acid concentrations [34]. These organic acids can mobilize less accessible phosphorus minerals in the soil [46], thereby fostering the growth of oligotrophic groups, like Chloroflexi and Alphaproteobacteria (Figure 5b) [47]. These groups can store absorbed carbon within cells under adverse conditions [48], ultimately enhancing soil SOC, stability, and TP accumulation. The incorporation of cover crops into fields leads to a shift in soil fungal communities, favoring Ascomycetes, particularly the Sordariomycetes species (Figure 5c,d), consistent with the current research [49]. Sordariomycetes play a crucial role in decomposing organic matter residues following the return of cover crops to the field [50]. Moreover, the enhancement of soil fertility leads to the convergence of fungal communities and reduces fungal abundance and intra-species interactions [49]. The enrichment of N in the HV treatment promoted Chytridiomycetes (Figure 5c), which in turn positively affected the utilization of diverse nitrogen sources [51,52].

Network complexity, a pivotal attribute of microbial communities, underpins soil multifunctionality. Our findings show that link density within the bacteria network was enhanced under the HV and RP treatment (Figure 6b). In essence, the incorporation of cover crops enhances the complexity of bacterial co-occurrence networks. Specifically, soil microbial communities under resource-acquisitive cover crop treatments are predominantly eutrophic, with soil microbes mediating network stabilization through rapidly available nitrogen. Conversely, the network in the RP treatment exhibited the highest level of complexity (Figure 6b,c). This phenomenon can be explained by the findings of Zhang et al., which indicate that low nitrogen addition rates enhance microbial biomass and activity by increasing nutrient availability, potentially shifting species interactions from mutualistic to competitive and leading to a more complex microbial network [53].

Investigating synergistic relationships among soil microorganisms is essential for deciphering soil ecosystem dynamics. Within these ecosystems, soil microbial networks exhibit both positive and negative correlations among species [35,54]. In this study, in the resource-acquisitive (HV) treatment, the proportion of negative correlations was lower than that in the resource-conservative (RP) treatment, suggesting a reduction in intra-community competition (Table 2). While negative correlations were present in the soil bacterial and fungal communities, they were invariably less frequent than positive interactions (Table 2). The significant release of readily decomposable nitrogen and carbon sources from resource-acquisitive cover crops, once returned to the field, supplies stable and ample nutrients for microbial growth [55]. Especially with excess nitrogen sources, microorganisms are prone to collaborate, promoting positive interactions, like symbiosis and mutualism [41,56]. A previous study showed that increased resource availability can broaden shared ecological niches and reduce interspecies competition [57]. This can explain why the proportion of negative correlations in the HV treatment was lower (Table 2). Conversely, an increase in negative correlations under the resource-conservative (RP) treatment suggests intensified intra-community competition (Table 2). Verdú et al. underscored that in multispecies systems, the structure of competitive relationships within complex networks dictated the system’s competitive dynamics [58]. Specifically, the organic carbon in resource-conservative cover crop residues is largely inert and resistant to rapid microbial degradation due to a high C/N ratio and complex organic matter presence [59]. Microbial communities compete for scarce resources via diverse mechanisms. Some species may be inhibited or outcompeted due to slow adaptation to environmental changes [60], intensifying competitive interactions and exacerbating resource depletion. Notably, despite intensified microbial competition, our results show an increase in bacterial species abundance (Figure 3a) and a decrease in fungal species abundance (Figure 3b). This phenomenon could be due to ecological niche differentiation among microorganisms, adapting to different nutrient sources and metabolic pathways in nutrient-poor environments [61,62]. For example, bacteria can secure nutrients via nitrogen fixation and the decomposition of complex organic matter [63]. This diverse resource utilization can alter species abundance, leading to increased bacterial species richness [64]. In contrast, fungi, typically necessitating greater nutrient availability and more consistent organic matter inputs, encounter difficulties in proliferating under nutrient-depleted conditions, resulting in a diminished abundance of fungal species [40].

## 5. Conclusions

This study presents a comprehensive pathway diagram illustrating the impact of cover crops with varying root traits on sweet potato yield, based on changes in the soil properties and microbial characteristics. Compared to winter fallowing, the resource-conservative cover crop primarily enhanced crop yield, significantly altering the diversity and community structure of the soil bacteria and improving the soil nutrient content. Although the yield increase from the resource-acquisitive cover crops was not statistically significant, there were notable differences in the microbial community structure, and the network structure showed greater stability. The introduction of suitable cover crop rotations to modulate a microbial network structure holds potential for enhancing the crop yield.

## Figures and Tables

**Table 1:** Root traits of two distinct cover crops.

Root Traits	D (mm)	V (cm^3^)	SA (cm^2^)	T	L (cm)	CC (mg/g)	NC (mg/g)	C/N
HV	1.03 ± 0.12 a	9.69 ± 1.64 a	320.44 ± 69.54 a	10,295.00 ± 685.00 a	1057.26 ± 86.40 a	338.27 ± 25.32 a	17.78 ± 0.52 a	19.01 ± 0.92 a
RP	1.24 ± 0.07 b	7.43 ± 0.30 b	240.60 ± 29.80 b	7865.00 ± 1131.00 b	551.64 ± 52.06 b	321.17 ± 19.76 a	3.91 ± 0.08 b	82.17 ± 3.66 b

Notes: HV, hairy vetch; RP, rapeseed. D, root diameter; C/N, root carbon-to-nitrogen ratio; CC, root carbon content; NC, root nitrogen content; V, root volume; SA, root surface area; T, number of root tips; L, root length. Different letters within the same row indicate significant differences among the treatments (*p* < 0.05).

**Table 2:** Topological characteristics of soil microbial co-occurrence networks.

	Soil Bacteria			Soil Fungi		
	CK	HV	RP	CK	HV	RP
Number of nodes	280	293	300	82	78	72
Number of edges	1024	1221	1304	494	484	352
Average degree	3.66	4.17	4.35	6.02	6.21	4.89
Linkage density	7.31	8.33	8.69	12.05	12.41	9.78
Positive	737 (71.94%)	816 (66.85%)	789 (60.48%)	332 (67.2%)	287(59.3%)	190(54.0%)
Negative	287 (28.06%)	405 (33.15%)	515 (39.52%)	162 (32.8%)	197 (40.7%)	162(46.0%)

## Data Availability

The original contributions presented in this study are included in the article. Further inquiries can be directed to the corresponding authors.

## Appendix A

**Table A1:** Differences in soil properties among cover crop treatments tested using LSD post hoc test.

	CK	HV	RP
pH	4.79 ± 0.12 bc	4.74 ± 0.13 c	4.95 ± 0.06 a
SOC (g/kg)	13.12 ± 0.67 b	13.35 ± 1.47 b	15.25 ± 0.63 a
TN (g/kg)	1.16 ± 0.07 a	1.18 ± 0.03 a	1.28 ± 0.02 a
TP (g/kg)	0.69 ± 0.04 b	0.69 ± 0.06 b	0.83 ± 0.04 a
AN (mg/kg)	123.65 ± 7.77 b	137.42 ± 7.33 a	133.78 ± 8.43 a
AP (mg/kg)	42.25 ± 16.51 a	45.75 ± 10.82 a	53.13 ± 10.56 a
BG (nmol/h/g)	11.94 ± 1.44 a	11.20 ± 1.17 a	13.57 ± 2.63 a
XYL (μmol/h/g)	5.56 ± 0.48 a	5.82 ± 1.39 a	5.81 ± 0.55 a
CB (nmol/h/g)	36.56 ± 10.85 b	38.03 ± 13.53 b	58.08 ± 5.21 a
NAG (μmol/h/g)	6.77 ± 2.72 a	6.40 ± 1.13 a	7.10 ± 1.45 a
LAP (μmol/h/g)	0.33 ± 0.10 b	0.44 ± 0.11 ab	0.62 ± 0.10 a
ACP (nmol/h/g)	89.48 ± 7.37 b	100.99 ± 8.55 ab	107.87 ± 6.90 a

Notes: Different letters within the same row indicate significant differences among the treatments.
